# Translation and preliminary validation of a Chinese version of a short dry eye symptom questionnaire

**DOI:** 10.3389/fmed.2026.1768373

**Published:** 2026-04-10

**Authors:** Juan Gui, Youran Cai, Xuyang Xu, Yiran Meng, Yi Du, Wenjing Luo

**Affiliations:** 1 Department of Ophthalmology, The First Affiliated Hospital of Guangxi Medical University, Nanning, China; 2 Department of Neurology, The First Affiliated Hospital of Guangxi Medical University, Nanning, China

**Keywords:** Chinese population, dry eye disease, Ocular Surface Disease Index, psychometric validation, screening, symptom questionnaire

## Abstract

**Objective:**

To translate a short dry eye symptom questionnaire into Chinese and evaluate its psychometric properties and screening performance for Ocular Surface Disease Index-defined symptomatic dry eye.

**Methods:**

The questionnaire was translated by forward-backward translation. In an anonymous online survey, 525 adults completed the Chinese short questionnaire and the Ocular Surface Disease Index. The original instrument includes two symptom-frequency items and one prior clinical diagnosis item; psychometric analyses used the summed dryness and irritation score. Ocular Surface Disease Index total scores were recalculated using the original algorithm, excluding “not applicable” responses from the denominator. Internal consistency was assessed with Cronbach's alpha, and convergent validity with Spearman correlations against Ocular Surface Disease Index total and domain scores. Receiver operating characteristic analysis used Ocular Surface Disease Index-defined symptomatic dry eye, defined as a score of 13 or greater, as the reference standard. The cutoff was selected by maximizing the Youden index.

**Results:**

All 525 questionnaires were retained. The median age was 32 years (interquartile range, 23–42), and 286/491 participants with valid sex coding (58.2%) were female. The median Ocular Surface Disease Index total score was 15.0 (interquartile range, 6.82–25.00), and 299/525 participants (57.0%) had Ocular Surface Disease Index-defined symptomatic dry eye. Internal consistency for the two symptom-frequency items was acceptable (Cronbach's alpha = 0.758). The two-item symptom sum correlated moderately with the Ocular Surface Disease Index total score (Spearman's rho = 0.669, *P* < 0.001), symptom domain (rho = 0.612), function domain (rho = 0.509), and environmental trigger domain (rho = 0.567; all *P* values < 0.001). The area under the receiver operating characteristic curve was 0.791. The practical cutoff was a two-item symptom sum of 4 or greater, with sensitivity of 89.3%, specificity of 56.2%, and overall agreement of 75.0% (Cohen's kappa = 0.472).

**Conclusion:**

The Chinese short questionnaire showed acceptable internal consistency, convergent validity, and moderate screening performance for Ocular Surface Disease Index-defined symptomatic dry eye. Its brevity supports symptom screening, but it should not be interpreted as a standalone diagnostic instrument for clinically confirmed dry eye disease.

## Introduction

Dry eye disease is a common, multifactorial disease of the ocular surface. It is characterized by loss of tear film homeostasis and is accompanied by ocular symptoms, in which tear film instability, hyperosmolarity, ocular surface inflammation and damage, and neurosensory abnormalities may play etiologic roles (1, 2). The reported prevalence varies widely across populations, including in China (2, 3). Dry eye symptoms can impair visual function, reduce quality of life, and are associated with depression, anxiety, and poor sleep (4–6). Early recognition of symptomatic individuals is therefore clinically relevant.

Because symptoms are central to the diagnosis and assessment of dry eye disease, patient-reported questionnaires are widely used in both clinical practice and research (7). The Ocular Surface Disease Index is one of the most commonly used instruments. It includes 12 items covering ocular symptoms, vision-related function, and environmental triggers, and it has shown good psychometric properties (8). In China, a Chinese version of the Ocular Surface Disease Index has also been used in validation research and clinical studies (9). However, longer questionnaires may increase respondent burden and may be less practical in rapid screening settings.

A short questionnaire based on prior clinical diagnosis and two symptom-frequency items, dryness and irritation, was introduced by Schaumberg et al. (10) in a large epidemiologic study of United States women. This short instrument was later clinically validated and shown to have acceptable repeatability (11). Its main advantage is brevity. However, to our knowledge, a Chinese version of this short questionnaire has not been formally evaluated.

The aim of the present study was to translate this short dry eye symptom questionnaire into Chinese and to perform a preliminary psychometric evaluation in a Chinese online adult sample. We focused on internal consistency, convergent validity, and screening performance against an Ocular Surface Disease Index-based symptom reference standard.

## Materials and methods

### Translation procedure

A forward-backward translation process was used to develop the Chinese version of the short dry eye symptom questionnaire. First, a bilingual translator whose native language was Chinese translated the questionnaire from English into Chinese. Second, an independent bilingual translator, blinded to the original version, back-translated the Chinese version into English. Third, discrepancies between the original and back-translated versions were reviewed by the research team and resolved by consensus. Finally, the Chinese wording was refined for conceptual equivalence and readability.

To avoid copyright concerns, the translated questionnaire is not reproduced in the manuscript. It is available from Dr. Yi Du, one of the corresponding authors, upon reasonable request for academic use.

### Measures

The original short questionnaire contains three items: prior clinical diagnosis of dry eye syndrome, frequency of ocular dryness, and frequency of ocular irritation (10, 11). In the original framework, dry eye syndrome is defined as either a prior clinical diagnosis or severe symptoms, with severe symptoms defined as both dryness and irritation being reported as often or constantly (10).

In the present study, all three original items were retained in the Chinese version to preserve content equivalence with the source instrument. For psychometric analyses of current symptom burden, we used the summed score of the dryness and irritation items. These two items were each coded from 1 to 4 and summed to produce a two-item symptom score ranging from 2 to 8. The prior clinical diagnosis item was retained for descriptive reporting and for comparison with the original questionnaire framework, but it was not included in the internal consistency analysis because it reflects diagnostic history rather than current symptom frequency.

All participants also completed the Ocular Surface Disease Index. The Chinese version used in this study followed the version reported by Lin et al. (9). The Ocular Surface Disease Index includes 12 items covering ocular symptoms, vision-related function, and environmental triggers (8). Each applicable item is scored from 0 to 4, and the total score is calculated as 25 × (sum of answered item scores ÷ number of answered items), yielding a score from 0 to 100 (8). For items 6 to 12, responses indicating that the activity or exposure did not occur during the previous week were treated as not applicable and were excluded from the denominator. For descriptive analyses, recalculated Ocular Surface Disease Index total scores were grouped as normal (<13), mild (13 to <23), moderate (23 to <33), and severe (≥33), consistent with commonly used severity bands and with the binary reference threshold of 13 or greater for symptomatic dry eye (12). For domain-specific analyses, domain scores were derived from their constituent items using the same 0–100 scaling approach.

### Participants and data handling

Participants were recruited over a 10-day period, from June 24 to July 3, 2022, through a WeChat network connected to ophthalmologists in Guangxi, China.

For the present reanalysis, the analytic dataset was rebuilt directly from the raw spreadsheet. All 525 returned records were retained for the primary psychometric analyses if the questionnaire items required for scoring were available. Age and sex were used only in subgroup analyses and were not required for inclusion in the primary analytic sample. Because a minority of sex entries contained unusable free-text strings, only exact male and female codes were treated as valid for sex-based subgroup analyses.

This sample should be regarded as a convenience sample obtained through online self-selection. It was not designed to be representative of the general Chinese adult population or of clinic-based dry eye populations.

### Statistical analysis

Statistical analyses were performed in Python 3.13.5 using pandas 2.2.3, NumPy 2.3.5, SciPy 1.17.0, and scikit-learn 1.8.0. Continuous variables are presented as medians and interquartile ranges, and categorical variables as counts and percentages.

Internal consistency of the two symptom-frequency items was assessed using Cronbach's alpha. Group differences in the two-item symptom score across Ocular Surface Disease Index severity categories were evaluated using the Kruskal–Wallis test. Convergent validity was assessed using Spearman rank correlation coefficients between the short questionnaire and the Ocular Surface Disease Index total score and domain scores.

Screening performance was evaluated by receiver operating characteristic analysis using Ocular Surface Disease Index-defined symptomatic dry eye, defined as a total score of 13 or greater, as the reference standard. The practical cutoff was selected by maximizing the Youden index, and agreement was summarized using overall agreement and Cohen's kappa.

To examine robustness using the existing dataset, we also performed split-sample, restricted sensitivity, and exploratory subgroup analyses. First, a random split-sample analysis was conducted using a 70:30 derivation-validation design, and the cutoff derived in the derivation subset was applied unchanged to the validation subset. Second, a restricted sensitivity analysis excluded participants with any positive general-condition screening item. Third, exploratory subgroup analyses were conducted according to sex and age.

## Results

### Participant characteristics

A total of 525 survey responses were available in the raw spreadsheet. After rebuilding the analytic dataset directly from the raw data, all 525 records were retained for the primary psychometric analyses. The median age was 32 years (interquartile range, 23–42 years). Among participants with valid sex coding, 286/491 (58.2%) were female and 205/491 (41.8%) were male. A prior clinical diagnosis of dry eye was reported by 60/525 participants (11.4%). According to the original questionnaire definition, 26/525 participants (5.0%) had severe symptoms and 76/525 (14.5%) met the original questionnaire-defined dry eye syndrome criterion.

The median Ocular Surface Disease Index total score was 15.0 (interquartile range, 6.82–25.00). A total of 299/525 participants (57.0%) had Ocular Surface Disease Index-defined symptomatic dry eye. Using recalculated Ocular Surface Disease Index total scores, 226 participants were classified as normal, 155 as mild, 100 as moderate, and 44 as severe. Participant characteristics and questionnaire summary scores are presented in Table 1.

**Table 1:** Participant characteristics and questionnaire scores.

Characteristic	Value
Primary psychometric sample size	525
Age available, *n*	521
Valid coded sex, *n*	491
Invalid or unusable sex entries, *n*	34
Age, median (IQR), years	32 (23–42)
Age ≤ 30 years, *n* (%) among available age	248 (47.6%)
Age 31–40 years, *n* (%) among available age	119 (22.8%)
Age 41–50 years, *n* (%) among available age	101 (19.4%)
Age >50 years, *n* (%) among available age	53 (10.2%)
Female, *n* (%) among valid coded sex	286 (58.2%)
Male, *n* (%) among valid coded sex	205 (41.8%)
Prior clinical dry eye diagnosis, *n* (%)	60 (11.4%)
Original severe symptoms, *n* (%)	26 (5.0%)
Original questionnaire-defined dry eye syndrome, *n* (%)	76 (14.5%)
Two-item symptom sum, median (IQR)	4 (3–4)
Ocular Surface Disease Index total score, median (IQR)	15.0 (6.82–25.00)
Ocular Surface Disease Index normal (<13), *n* (%)	226 (43.0%)
Ocular Surface Disease Index mild (13 to <23), *n* (%)	155 (29.5%)
Ocular Surface Disease Index moderate (23 to <33), *n* (%)	100 (19.0%)
Ocular Surface Disease Index severe (≥33), *n* (%)	44 (8.4%)
Ocular Surface Disease Index-defined symptomatic dry eye, *n* (%)	299 (57.0%)

### Short questionnaire scores across Ocular Surface Disease Index severity categories

Across the recalculated Ocular Surface Disease Index severity categories, the median two-item symptom sum increased from 3 (interquartile range, 2–4) in the normal group to 5 (interquartile range, 4–6) in the severe group. The difference across severity groups was statistically significant (Kruskal-Wallis H = 202.16, P < 0.001).

For the individual symptom items, the median dryness score was 2 (interquartile range, 2–2) overall, and the median irritation score was 2 (interquartile range, 1–2) overall. In the severe Ocular Surface Disease Index group, the median dryness score was 3 (interquartile range, 2–3) and the median irritation score was 2 (interquartile range, 2–3). Detailed distributions of the two-item symptom sum and the two individual symptom items across Ocular Surface Disease Index severity categories are shown in Table 2.

**Table 2:** Short questionnaire scores across Ocular Surface Disease Index severity categories.

Ocular Surface Disease Index category	*n*	Two-item symptom sum, median (IQR)	Dryness item, median (IQR)	Irritation item, median (IQR)
Normal (<13)	226	3 (2–4)	2 (1–2)	1 (1–2)
Mild (13 to <23)	155	4 (4–4)	2 (2–2)	2 (2–2)
Moderate (23 to <33)	100	4 (4–5)	2 (2–3)	2 (2–2)
Severe (≥33)	44	5 (4–6)	3 (2–3)	2 (2–3)
Overall	525	4 (3–4)	2 (2–2)	2 (1–2)

Kruskal–Wallis test for the two-item symptom sum across Ocular Surface Disease Index severity groups: *H* = 202.16, *P* < 0.001.

### Internal consistency reliability

Internal consistency was assessed for the two symptom-frequency items only, because the prior clinical diagnosis item reflects diagnostic history rather than current symptom frequency. The two-item Chinese short questionnaire showed acceptable internal consistency, with a Cronbach's alpha of 0.758.

### Convergent validity

The two-item symptom sum showed a moderate positive correlation with the Ocular Surface Disease Index total score (Spearman's rho = 0.669, *P* < 0.001). It also correlated with the Ocular Surface Disease Index symptom domain (rho = 0.612, *P* < 0.001), function domain (rho = 0.509, *P* < 0.001), and environmental trigger domain (rho = 0.567, *P* < 0.001).

The dryness item correlated with the Ocular Surface Disease Index total score (rho = 0.593, *P* < 0.001), symptom domain (rho = 0.543, *P* < 0.001), function domain (rho = 0.461, *P* < 0.001), and environmental trigger domain (rho = 0.489, *P* < 0.001). The irritation item correlated with the Ocular Surface Disease Index total score (rho = 0.605, *P* < 0.001), symptom domain (rho = 0.565, *P* < 0.001), function domain (rho = 0.442, *P* < 0.001), and environmental trigger domain (rho = 0.526, *P* < 0.001). The full correlation matrix is presented in Table 3.

**Table 3:** Convergent validity of the Chinese short dry eye symptom questionnaire.

Predictor	Outcome	Spearman's rho	*P* value
Two-item symptom sum	Ocular Surface Disease Index total score	0.669	<0.001
Two-item symptom sum	Ocular Surface Disease Index symptom domain	0.612	<0.001
Two-item symptom sum	Ocular Surface Disease Index function domain	0.509	<0.001
Two-item symptom sum	Ocular Surface Disease Index environmental trigger domain	0.567	<0.001
Dryness item	Ocular Surface Disease Index total score	0.593	<0.001
Dryness item	Ocular Surface Disease Index symptom domain	0.543	<0.001
Dryness item	Ocular Surface Disease Index function domain	0.461	<0.001
Dryness item	Ocular Surface Disease Index environmental trigger domain	0.489	<0.001
Irritation item	Ocular Surface Disease Index total score	0.605	<0.001
Irritation item	Ocular Surface Disease Index symptom domain	0.565	<0.001
Irritation item	Ocular Surface Disease Index function domain	0.442	<0.001
Irritation item	Ocular Surface Disease Index environmental trigger domain	0.526	<0.001

**Figure 1:**
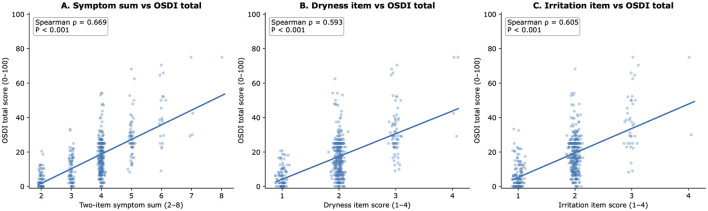
Correlations between the Chinese short dry eye symptom questionnaire and Ocular Surface Disease Index total scores. **(A)** shows the association between the two-item symptom sum (dryness + irritation) and the Ocular Surface Disease Index total score. **(B)** shows the association between the dryness item and the Ocular Surface Disease Index total score. **(C)** shows the association between the irritation item and the Ocular Surface Disease Index total score.

**Figure 2:**
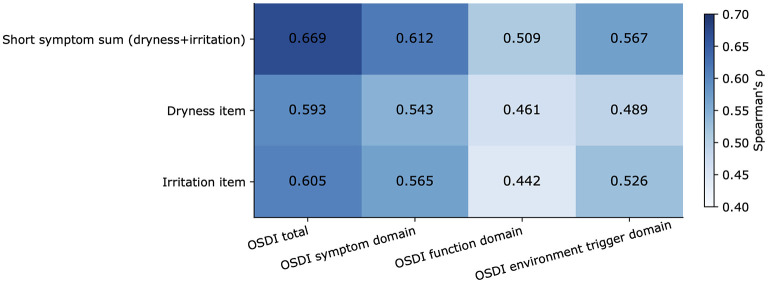
Domain-specific convergent validity of the Chinese short dry eye symptom questionnaire. Heatmap of Spearman correlation coefficients between the two-item symptom sum, the dryness item, and the irritation item and the Ocular Surface Disease Index total score, symptom domain, function domain, and environmental trigger domain.

The associations between the short questionnaire variables and the **Ocular Surface Disease Index total score** are shown in Figure 1. The overall pattern of domain-specific correlations is summarized in Figure 2.

### Screening performance for Ocular Surface Disease Index-defined symptomatic dry eye

**Figure 3:**
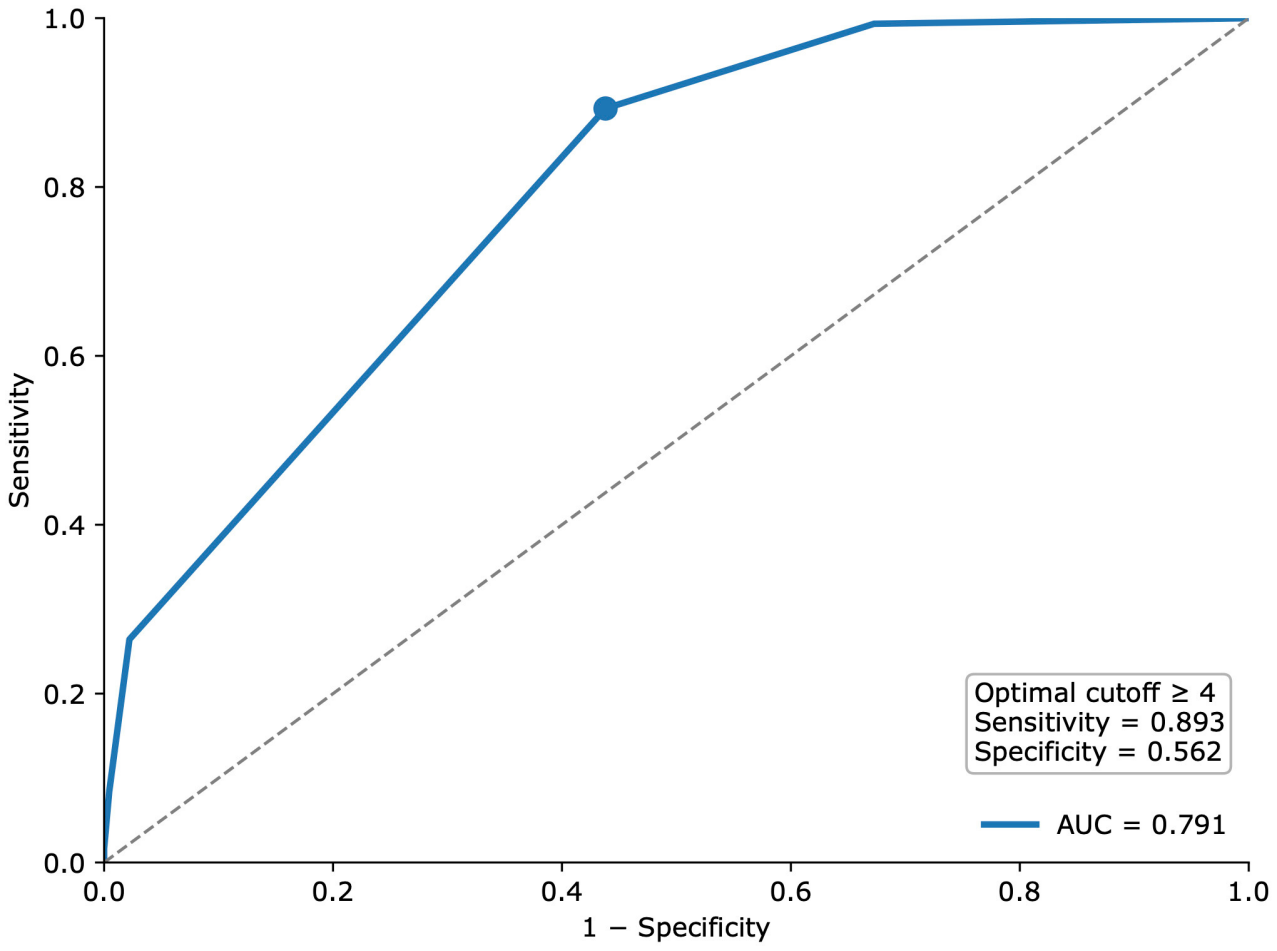
Receiver operating characteristic curve for the Chinese short dry eye symptom questionnaire using Ocular Surface Disease Index-defined symptomatic dry eye, defined as a score of 13 or greater, as the reference standard. The area under the receiver operating characteristic curve was 0.791. The practical cutoff was a two-item symptom sum of 4 or greater.

Receiver operating characteristic analysis was performed using Ocular Surface Disease Index-defined symptomatic dry eye, defined as a score of 13 or greater, as the reference standard. The area under the receiver operating characteristic curve was 0.791. The optimal cutoff identified by the Youden index was a two-item symptom sum of 4, which was adopted as the practical cutoff. The receiver operating characteristic curve is shown in Figure 3.

At the practical cutoff of 4 or greater, sensitivity was 0.893 and specificity was 0.562. Using this threshold, 366 participants screened positive on the short questionnaire, compared with 299 participants classified as positive by the Ocular Surface Disease Index reference standard. Overall agreement was 0.750, and Cohen's kappa was 0.472. The screening performance results are summarized in Table 4.

**Table 4:** Screening performance of the Chinese short dry eye questionnaire for Ocular Surface Disease Index-defined symptomatic dry eye.

Analysis set	*n*	Reference positives, *n* (%)	Area under the curve	Practical cutoff	Sensitivity	Specificity	Overall agreement	Cohen's kappa
Primary sample	525	299 (57.0%)	0.791	≥4	0.893	0.562	0.750	0.472
Restricted sensitivity set^*^	399	209 (52.4%)	0.783	≥4	0.871	0.574	0.729	0.450
Derivation subset (70%)	367	209 (56.9%)	0.795	≥4	0.909	0.557	0.757	0.485
Validation subset (30%)	158	90 (57.0%)	0.784	≥4	0.856	0.574	0.734	0.442

OSDI, ocular surface disease index.

^*^Restricted sensitivity set: participants with all four general-condition screening items negative, namely current ocular disease, prior ocular surgery, dry eye-related systemic disease, and current dry eye treatment. Reference positives were defined as Ocular Surface Disease Index-defined symptomatic dry eye, based on a recalculated Ocular Surface Disease Index total score of 13 or greater. The practical cutoff was selected by maximizing the Youden index.

### Additional robustness analyses

In the split-sample analysis, the practical cutoff remained 4 or greater in both subsets. In the derivation subset, the area under the receiver operating characteristic curve was 0.795, with sensitivity of 0.909 and specificity of 0.557. In the validation subset, the area under the receiver operating characteristic curve was 0.784, with sensitivity of 0.856 and specificity of 0.574.

In the restricted sensitivity analysis excluding participants with any positive general-condition screening item, the area under the receiver operating characteristic curve was 0.783, with sensitivity of 0.871 and specificity of 0.574 at the same cutoff. Exploratory subgroup analyses showed the same practical cutoff of 4 or greater across sex and age strata, with area under the receiver operating characteristic curve values ranging from 0.743 to 0.840. These robustness analyses are summarized in Table 4.

## Discussion

In this study, we translated a short dry eye symptom questionnaire into Chinese and performed a preliminary psychometric evaluation in an online adult sample. The Chinese version showed acceptable internal consistency, moderate convergent validity with the Ocular Surface Disease Index, and moderate screening performance for Ocular Surface Disease Index-defined symptomatic dry eye.

A key point is that the original instrument contains three items, including prior clinical diagnosis, dryness frequency, and irritation frequency (10, 11). In the original framework, questionnaire-defined dry eye syndrome is based on either prior diagnosis or severe symptoms, with severe symptoms defined as both dryness and irritation being reported as often or constantly (10). In contrast, our main psychometric analyses focused on the summed score of the two symptom-frequency items, because this two-item component reflects current symptom burden and is suitable for internal consistency and receiver operating characteristic analyses. Separating the original three-item framework from the two-item symptom score is important for accurate interpretation.

Another central issue is the choice of reference standard. We did not perform objective ocular surface examinations, and our receiver operating characteristic analysis used Ocular Surface Disease Index-defined symptomatic dry eye, defined as a score of 13 or greater, as the comparator. Therefore, our results do not show that the Chinese short questionnaire detects clinically confirmed dry eye disease. Rather, they show that the questionnaire performs reasonably well in screening for symptom-defined dry eye. This distinction matters and should be stated clearly, because symptom questionnaires and clinical signs are often only modestly correlated in dry eye research (7).

The specificity at the practical cutoff was modest. This means that the instrument is better suited to initial symptom screening than to confirmatory diagnosis. In practical terms, a positive result on this questionnaire should prompt further evaluation rather than establish dry eye disease by itself. This limitation is especially relevant in populations with a low pretest probability of disease, where false positives may be more frequent. The present instrument is therefore better understood as a brief symptom screener than as a diagnostic tool.

The domain-specific analyses strengthened construct validity. The two-item symptom sum correlated most strongly with the Ocular Surface Disease Index total score and the symptom domain, while correlations with the function and environmental trigger domains were somewhat lower. This pattern is plausible. The short questionnaire directly measures current symptom frequency, whereas the Ocular Surface Disease Index includes broader functional and environmental constructs (8). The findings support the view that the short questionnaire captures the symptom core of dry eye, but not the full breadth of disability assessed by the Ocular Surface Disease Index.

Compared with other short symptom instruments used in Chinese dry eye research, such as the Standard Patient Evaluation of Eye Dryness, this questionnaire has a clear practical advantage: it is even briefer (13). That is useful when rapid symptom screening is needed. The trade-off is narrower content coverage than longer instruments such as the Ocular Surface Disease Index and other validated Chinese dry eye questionnaires, including the Symptom Assessment in Dry Eye (SANDE) questionnaire (9, 13). In addition, short instruments are not necessarily interchangeable. Previous work comparing dry eye questionnaires has shown meaningful differences in scale structure and score behavior (14). The present questionnaire should therefore be viewed as a simple symptom screener, not as a replacement for more comprehensive symptom profiling or for clinical diagnosis.

Several limitations should be emphasized. First, this was an online convenience sample recruited through a WeChat network related to ophthalmology. Selection bias and healthcare-associated sampling bias are therefore likely. The sample should not be considered representative of the general Chinese adult population or of clinic-based dry eye populations. Second, no objective ocular surface tests were collected, so we could not validate the questionnaire against clinical signs or a clinical diagnostic standard. Third, test-retest reliability was not assessed because the survey was anonymous and cross-sectional. This remains an important gap for future work, especially because repeatability was examined in the original clinical validation study (11). Finally, although the split-sample and restricted sensitivity analyses supported the stability of the practical cutoff, external validation in clinic-based cohorts remains necessary.

## Conclusion

The Chinese short dry eye symptom questionnaire showed acceptable internal consistency and convergent validity, and it demonstrated moderate screening performance for Ocular Surface Disease Index-defined symptomatic dry eye. Its brevity makes it suitable for rapid symptom screening, but it should not be interpreted as a standalone diagnostic instrument for clinically confirmed dry eye disease.

## Data Availability

The raw data supporting the conclusions of this article will be made available by the authors, without undue reservation.
